# Differential regulatory strategies for spring and autumn migrations in Palearctic-Indian songbird migrants

**DOI:** 10.3389/fphys.2022.1031922

**Published:** 2022-09-29

**Authors:** Vinod Kumar, Aakansha Sharma, Vatsala Tripathi, Sanjay Kumar Bhardwaj

**Affiliations:** ^1^ IndoUS Center in Chronobiology, Department of Zoology, University of Delhi, Delhi, India; ^2^ IndoUS Center in Chronobiology, Department of Zoology, University of Lucknow, Lucknow, India; ^3^ Department of Zoology, Dyal Singh College, University of Delhi, Delhi, India; ^4^ Department of Zoology, CCS University, Meerut, India

**Keywords:** migration, seasonal, songbird, spring vs. autumn, metabolism

## Introduction

Birds, like most if not all long-lived species, show cyclic behavior and physiology. They exhibit daily cycles in activity-rest, sleep-wake, feeding-starvation and high-low body temperature, and annual (seasonal) cycles in growth, migration, hibernation, reproduction and molt. These are the products of endogenous circadian (Latin: *circa* = about; *dies* = day) and circannual (Latin: *circa* = about; *annum* = year) timers interacting with reliable environmental *Zeitgebers* (*zeit* = time, *geber* = giver; or time cue), e.g., changes in day length (= photoperiod), temperature, and/or feeding resources. For seasonal cycles, for example, the circannual timer and photoperiodic regulation seem mutually inclusive mechanisms for seasonal LHSs comprising the annual cycle of a bird species (Misra et al., 2004). It remains poorly understood though if the photoperiod changes directly or with its consistent interaction (synchronization) control changes in seasonal behavioral and physiological phenotypes during the annual itinerary of a species. However, the most accepted view is that endogenous circannual rhythm set the temporal window of a seasonal life-history state (LHS) during the year, and the photoperiod change regulates the physiological preparedness for the appropriate biological response characteristics of a LHS (Kumar et al., 2010). This can be more challenging for millions of songbirds that follow a rigid seasonal schedule, with migrations placed before and after the reproduction. This brief article aims to highlight in particular, the differential adaptive strategies that migrants employ during non-migratory and migratory LHSs, and during spring and autumn migrant periods, largely based on our research on Palearctic-Indian migratory buntings—the blackheaded bunting (*Emberiza melanocephala*) and redheaded bunting (*Emberiza bruniceps*).

## Photoperiod changes can reproduce the annual itinerary of a latitudinal avian migrant

A migrant’s annual itinerary includes molt, autumn migration, overwintering period, vernal migration and reproductive seasonal LHSs. These are identifiable by striking differences in birds’ behavioral and physiological phenotypes. For instance, the migrants maintain lean body mass except in the migratory period, and small reproductively inactive gonads except in the breeding season. With the transition from non-migrant to the migrant period, the birds show hyperphagia (increased foraging), fat accumulation in adipose and liver tissues to serve as flight fuel, and muscular hypertrophy for an enhanced flight endurance. There is also a drastic change in the behavior and physiology as exhibited in phase inversion of the 24-h pattern of activity behavior (from daytime to predominantly night-time activity) and olfaction and vision sensory systems linked with migration (Rastogi et al., 2011).

The timely departure gives time to explore feeding resources on the way and nesting resources at breeding grounds that enhance reproductive success. Post vernal equinox period, the overwintering birds begin to prepare for their northward migratory travel in response to increasing spring photoperiods (≥12 h daily light); the converse is true for the post autumn equinox period when birds respond to a decreasing photoperiod despite it being still close to the threshold for photoperiodic induction in spring. The ambient temperature also plays a crucial role in the development of the migration phenotype, as evidenced by transcriptional response to temperature in the development of spring migratory phenotype in captive redheaded buntings (Sur et al., 2020). The decision for migratory departure is, therefore, the outcome of the integration of environmental photoperiod and temperature with the migratory context (to breeding grounds in spring and to wintering areas in autumn) and the physiological state (pre-reproductive state in spring vs. post-reproductive state in autumn).

Seasonal LHSs can be faithfully reproduced in captive migratory buntings. Under short days mimicking a winter photoperiod, buntings maintain normal body mass and reproductively immature (inactive) gonads as well as responsivity to the photoperiodic induction. On exposure to long days (≥12 h, equal to or longer than the threshold photoperiod), the cascade of photoperiod-induced processes culminates into the development of spring migratory (copious subcutaneous fat deposition and *Zugunruhe—*intense nocturnal restlessness in captives) and reproductive (mature gonads) phenotypes (Misra et al., 2004; Rani et al., 2005, 2006; Malik et al., 2014; Trivedi et al., 2014; Singh et al., 2015). Prolonged long-day exposure leads to spontaneous regression, and birds become lean in body mass and day active again with regressed gonads. Subsequent response to a shorter photoperiod mimicking the late autumn day length, induces the autumn migratory phenotype (Singh et al., 2015; Sharma et al., 2018a).

## Neural and molecular correlates of migratory phenotype

The transition into a migratory phenotype requires changes at the regulatory (brain) and effector organ (liver, muscle) levels. Rastogi et al. (2011) showed that in parallel with daily behavioral cycles, there was a phase inversion in Fos-immunoreactivity (a marker of the neuronal activity) of olfactory and visual sensory circuits involved in orientation and navigation. The dorsomedial part of the mediobasal hypothalamus (not suprachiasmatic nucleus) seems to be the site of photoperiod-induced functional coupling of endogenous seasonal clock and behavioral outputs (e.g., *Zugunruhe*) relevant to the migration (Rastogi et al., 2013). At molecular level, a large number of hypothalamic cytoskeletal and calcium signaling (important for neurogenesis and maintenance of synaptic connections) genes were also upregulated in redheaded buntings exhibiting the photoperiod-induced migratory phenotype (Sharma et al., 2018b). The liver was also enriched with genes associated with intracellular protein transport, calcium ion transport and small GTPase-mediated signal transduction pathways during the migrant period (Sharma et al., 2018b). Interestingly, there was a molecular switch in genes associated with energy utilization being highly expressed during the day and night during non-migratory and migratory LHSs, respectively (Sharma et al., 2022).

## Differential strategies for spring and autumn migrations

**FIGURE 1:**
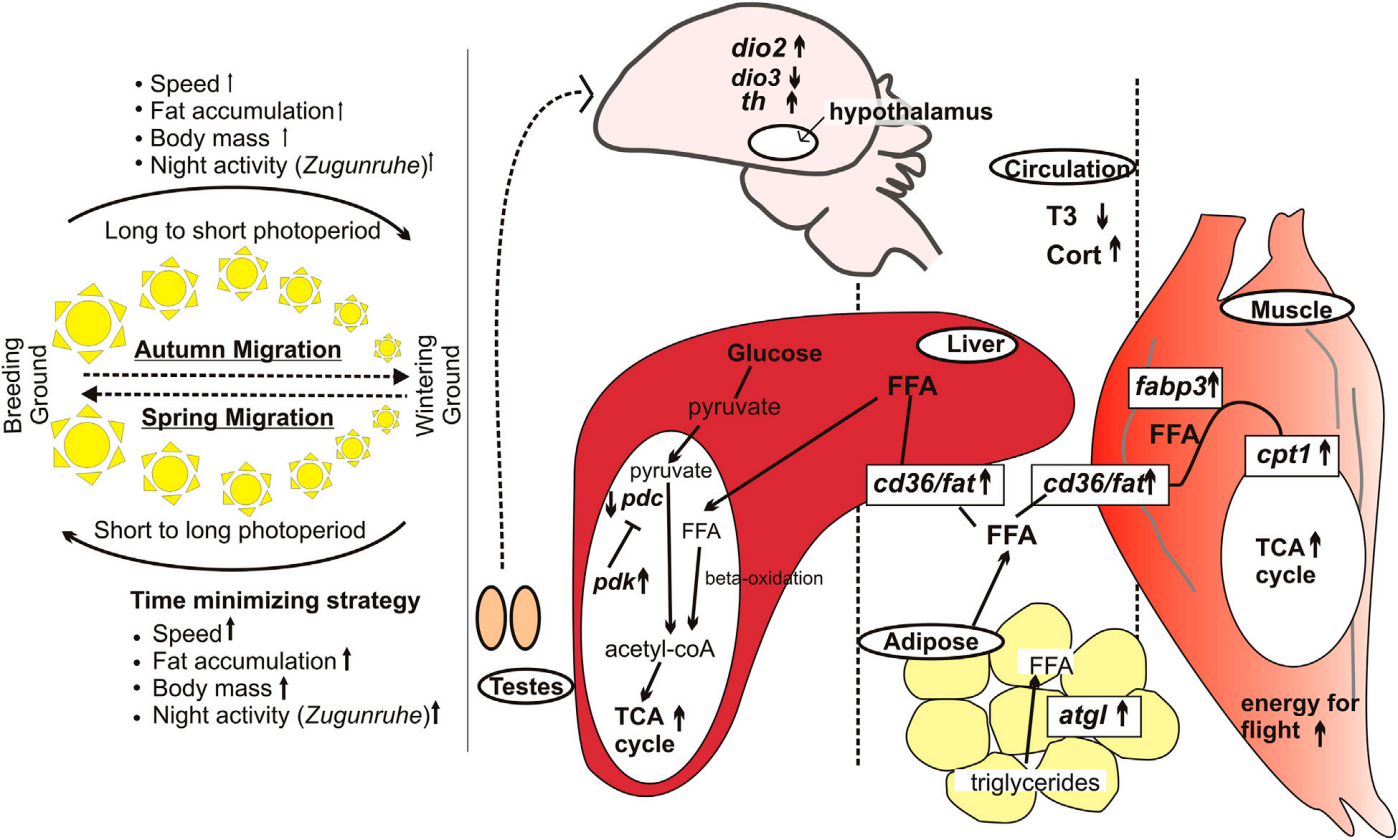
Differences in spring and autumn migrations, based on studies in captive migratory redheaded buntings. Left panel: Phenotypic changes associated with spring and autumn migrations. The thickness of arrows reflects differences in magnitude of the photostimulated response. The sun cartoons in the middle indicate the direction of photoperiod change. Right panel: Molecular changes in the hypothalamus, liver, muscle and adipose tissues in spring compared to the autumn migration. In spring, migrants show elevated plasma Cort and reduced T3 levels, and reciprocal switching of *dio2* and *dio3*, and increased *th* expression. The adipose triglycerides are converted into free fatty acids, which are up taken from circulation mediated by the enzymes and proteins encoded by *cd36*/*fat*, *fabp3* and *cpt1* genes. Hepatic *pdc* and *pdk* encoded enzymes regulate the fuel use. The arrow and the line ending with bar represent activation and inhibitory pathways, respectively. Upward arrows indicate upregulated gene expression in spring compared with that in the autumn migration (Sharma et al., 2018a; Sharma and Kumar 2019).

The two seasonal migrations placed before and after the breeding season differ in several ways. First is the difference in context: spring (or vernal) migration is for the timely arrival of migrating birds at the breeding grounds, and so it is at a faster pace with fewer stopovers (hence longer flight bouts) (Newton, 2007; Nilsson et al., 2013). Hence, with relatively less opportunity to re-fuel, spring migrants need to acquire a copious amount of fat before they start nocturnal flight (Sharma et al., 2018a; Sharma et al., 2018b; Sharma and Kumar, 2019) (Figure 1). There can also be a sex-dependent difference in the migratory drive since males need to reach early in order to define their territories and build nests, which are key determinants of a successful reproduction (Tryjanowski and Yosef, 2002). Although reproductively immature at the time of departure, gonadal tissues grow over the course of the vernal migratory journey and secrete hormones facilitating an immediate onset of reproductive behavior upon arrival at the breeding ground (Wingfield et al., 1990). By contrast, the autumn migration is to escape from harsh winter conditions and to allow recovery and repair post-reproduction on wintering grounds with rich feeding resources. Secondly, birds during two seasonal migrations differ in the photoperiodic state: they are sensitive and refractory to long day at the beginning of spring and autumn migrant periods, respectively. Thirdly, the migrating birds also face differences in the direction of photoperiod (and perhaps temperature) change; for example, the birds experience increasing and decreasing photoperiods during the spring and autumn travels, respectively.

### Hypothalamic control

Several recent studies have reported changes in hypothalamic gene expressions in migrant, compared to the non-migrant period. For example, the circadian clock gene (*per2*, *bmal1*) expressions showed alteration in their oscillatory waveform (24-h acrophase and amplitude) in redheaded buntings expressing photoperiod-induced transition from non-migratory to migratory LHS (Singh et al., 2015). Singh and Kumar (2017) also found increased amplitude and acrophase of *cry1* and *bmal1* (not *per2*) 24-h rhythms in the optic tectum and cerebellum of buntings expressing the *Zugunruhe*. Further, Sharma et al. (2018a) reported differential expression of thyroid hormone-responsive *dio2* and *dio3*, and light responsive *cry1*, *per2* and *adcyap1* genes between spring and autumn migrant periods. Consistent with the time-minimizing strategy and enhanced motivation, redheaded buntings almost doubled their *th* (tyrosine hydroxylase) expression during the photoperiod-induced spring migratory state (Sharma et al., 2018a) (Figure 1).

### Metabolic support

As in the hypothalamus, *per2* and *bmal1* clock genes showed alteration in their oscillatory waveform in liver of buntings with transition from non-migratory to the migratory LHS (Singh et al., 2015; Singh et al., 2018). The metabolic machinery seems modulated to support differential physiological requirements between spring and autumn migrant periods in buntings (Singh et al., 2018; Sharma and Kumar, 2019). Histological examination of the liver and adipose tissues revealed larger fat accumulation (shown by adipose cell area and hepatic fat droplets) during spring migratory state (Sharma and Kumar, 2019). There was an overall upregulation of fat utilization machinery during spring. Hepatic pyruvate dehydrogenase complex (PDC) and pyruvate dehydrogenase kinase (PDK) enzymes act antagonistically (PDK inactivates PDC) and regulate glycolytic cycle (conversion of pyruvate into acetyl-CoA) for the tricarboxylic acid cycle. A second substrate feeding to TCA cycle is by increased fatty acid oxidation. In simulated spring migrants, we found increased *pdk* and decreased *pdc* mRNA expressions in redheaded buntings (Sharma and Kumar, 2019). Increased fat utilization is evidenced by increased mRNA expression of genes coding for adipose tissue triglyceride lipase (*atgl*) in adipose tissues; and those coding for fatty acid translocase (*fat*/*cd36*), fatty acid binding protein (*fabp3*) and carnitine palmitoyl transferase 1 (*cpt1*) in the flight muscle. Enhanced conversion of triglycerides into free fatty acids can be up taken by flight muscles using FAT, FABP and CPT1 and be utilized by beta-oxidation for energy generation. Increased expressions of metabolic genes during migrant compared to non-migrant period, as well as during spring compared to autumn migrant period, suggest a robust seasonal metabolic plasticity in migratory buntings (Sharma and Kumar, 2019) (Figure 1).

### Role of gonads

A key difference lies in the gonadal state between two seasonal migrations. Gonad is in the preparatory and regression phases of its annual cycle before migration in spring and autumn, respectively. A recent redheaded bunting study demonstrated that the removal of testes affected the hypothalamic transcriptome but the effect was much larger in simulated autumn migrant period. The castrated buntings expressed reduced Z*ugunruhe* in autumn, compared to spring (Sharma et al., 2020). A large number of genes were also differentially expressed between intacts and castrates in autumn (62 genes), compared to the spring (37 genes). The differentially expressed genes in spring enriched G-protein coupled acetylcholine receptor signaling and signal transduction pathways, while those in autumn enriched largely the calcium signaling pathway (Sharma et al., 2020).

To sum up, the latitudinal migratory songbirds exhibit a significant seasonal difference in gene expressions between spring and autumn, concurrently with changes in behavior and physiology associated with migration. There are comprehensive changes in the olfaction, visual and hypothalamic neural circuits with the transition from non-migratory to migratory LHS. The songbird migrants utilize fat as major flight fuel supplied by adipose tissues *via* FFAs to support the migratory flight. The mechanisms underlying the generation and overall flux of energy involve oxidation of fatty acids in the liver and protein-mediated transport to the “working” muscles. The present discussion on neural and metabolic plasticities mediating differential seasonal responses provides molecular insights into the seasonal homeostasis at both regulatory and effector organ levels in long-lived species, possibly including humans.
